# Greater improvements in diet quality among overweight participants following a group-based commercial weight loss programme than those receiving support to lose weight in primary care

**DOI:** 10.1186/s12937-018-0370-x

**Published:** 2018-07-04

**Authors:** Gina L. Ambrosini, Ivonne Solis-Trapala, Amy L. Ahern, Nicholas R. Fuller, Christina Holzapfel, Hans Hauner, Ian D. Caterson, Susan A. Jebb

**Affiliations:** 1 School of Population and Global Health (M431), University of Western Australia, 35 Stirling Highway, Crawley, Perth, 6009 Western Australia Australia; 2 Medical Research Council Elsie Widdowson Laboratory, Cambridge, CB1 9NL England; 3 Institute for Applied Clinical Sciences, Keele University, Keele, Staffordshire, ST5 5BG England; 4 MRC Epidemiology Unit, University of Cambridge, Institute of Medical Sciences, Box 285, Cambridge, CB2 0QQ England; 5 The Boden Institute of Obesity, Nutrition, Exercise and Eating Disorders, Charles Perkins Centre, The University of Sydney, Sydney, NSW 2006 Australia; 6 Else Kroener-Fresenius-Center for Nutritional Medicine, Faculty of Medicine, Technical University of Munich, Munich, Germany; 7 Nuffield Department of Primary Care Health Sciences, University of Oxford, New Radcliffe House, Radcliffe Observatory Quarter, Woodstock Road, Oxford, OX2 6GG England

**Keywords:** Diet quality, Dietary change, Weight loss, Randomised controlled trial, Behavioural intervention

## Abstract

**Background:**

Relatively little is known about dietary changes and their relationships with weight change during behavioural weight loss interventions. In a secondary analysis of data from a multicentre RCT, we investigated whether greater improvements in diet would be achieved by overweight adults following a 12 month group-based commercial weight loss programme (CP) than those receiving standard care (SC) in primary practice, and if these dietary changes were associated with greater weight loss.

**Methods:**

Adults with a BMI 27–35 kg/m^2^ and >1 risk factor for obesity-related disorders were recruited in study centres in Australia and the UK during 2007–2008. Dietary intake and body weight were measured at baseline, 6 and 12 months. Linear mixed effects models compared mean changes in dietary macronutrient intake, fibre density and energy density over time between groups, and their relationships with weight loss.

**Results:**

The CP group demonstrated greater mean weight loss than the SC group at 6 months (3.3 kg, 95% CI: 2.2, 4.4) and 12 months (3.3 kg, 95% CI: 2.1, 4.5). Diet quality improved in both intervention groups at 6 and 12 months. However, the CP group (*n* = 228) achieved significantly greater mean reductions in energy intake (mean difference; 95% CI: − 503 kJ/d; − 913, − 93), dietary energy density (− 0.48 MJ/g; − 0.81, − 0.16), total fat (− 6.9 g/d; − 11.9, − 1.8), saturated fat (− 3.3 g/d; − 5.4, − 1.1), and significantly greater mean increases in fibre density (0.30 g/MJ; 0.15, 0.44) at 6 months than the SC group (*n* = 239). Similar differences persisted at 12 months and the CP group showed greater mean increases in protein density (0.65 g/MJ). In both groups, weight loss was associated with increased fibre density (0.68 kg per g/MJ, 95% CI: 0.08, 1.27) and protein density (0.26 kg per g/MJ, 95% CI: 0.10, 0.41).

**Conclusions:**

Following a group-based commercial program led to greater improvements in diet quality than standard care. Increases in dietary protein and fibre density were independently associated with weight loss in both behavioural weight loss interventions. Greater increases in protein and fibre density in the commercial program likely contributed to their greater weight loss.

**Trial registration:**

ISRCTN: ISRCTN85485463 Registered 03/08/2007 Retrospectively Registered.

**Electronic supplementary material:**

The online version of this article (10.1186/s12937-018-0370-x) contains supplementary material, which is available to authorized users.

## Background

Evidence from clinical trials shows that behavioural interventions aimed at helping individuals to acquire the knowledge and skills to change dietary intake, physical activity, and other weight-related behaviours can lead to successful weight loss [1]. Behavioural weight loss interventions typically encourage changes in dietary intake that result in reduced energy intake, as well as strategies to change behaviours such as self-monitoring, goal setting and implementation plans [2, 3]. However, relatively few behavioural weight loss interventions have detailed the changes that occur in dietary intake or their associations with weight change [4–8]. This information may help to identify the effective characteristics of interventions [9].

Behavioural interventions delivered in commercial group-based programmes usually result in greater weight losses than those led by primary care staff, which might be explained by greater reductions in energy intake and/or specific changes in diet quality [10, 11]. Here we report changes in dietary intake and test their relationships with weight loss using data from a 12 month, multicenter, randomised controlled trial (RCT) that compared weight loss after referral to free access to a commercial weight loss programme (CP) or weight management advice provided as standard care (SC) in a primary care setting [10]. In this secondary analysis of RCT data, we investigated changes in self-reported dietary energy density, macronutrient (total fat, saturated fat, protein, carbohydrate, sugars) and fibre intakes as indicators of diet quality or because of their associations (probable or confirmed) with body weight [12–20]. We have previously shown that participants randomised to the CP lost more weight than those receiving SC [10]. Accordingly, we hypothesised that participants who followed the CP would have achieved greater reductions in dietary energy density, total energy, total fat, saturated fat, total carbohydrate and sugar intake, and greater increases in dietary fibre and protein intake than the SC group at 6 and 12 months after baseline. We also examined which of these dietary changes were associated with the greatest weight loss at 6 and 12 months.

## Methods

### Study participants

Full details of the original trial (ISRCTN: 85485463; http://www.isrctn.com/ISRCTN85485463) have been published previously [10]. In brief, the trial was conducted through study centres in three countries (Australia, Germany and the UK) between September 2007 and November 2008. Trial participants were recruited from primary care practices.

The original trial (*n* = 772) compared weight change after randomisation to receive either referral to 12 months free access to a group-based commercial weight loss programme (CP; Weight Watchers®), or 12 months of standard care (SC; weight management advice defined by national treatment guidelines for each country and usually delivered by a practice nurse in a primary care setting). The study population consisted of adults who were moderately overweight or obese, with less severe comorbidities and at a low risk of treatment complications, making them suited to a commercial weight loss program setting. Eligible trial participants were adults with a body mass index (BMI) of 27–35 kg/m^2^ and at least one risk factor for obesity-related disorders, e.g. central adiposity, type 2 diabetes without insulin treatment, family history of type 2 diabetes, previous gestational diabetes, impaired glucose tolerance or impaired fasting glycaemia, mild to moderate dyslipidaemia, treatment for dyslipidaemia or hypertension [10]. Additional eligibility criteria and exclusion criteria for the trial are detailed elsewhere [10].

For the present analysis, trial data collected at baseline, 6 and 12 months after baseline were used. Estimated intakes of several dietary variables of key interest, i.e. dietary energy density, sugars, saturated fat, and comparable fibre intakes, were not available for participants recruited in Germany (*n* = 268), therefore only data from Australian (*n* = 268) and UK (*n* = 236) centres were included in these analyses, to ensure a consistent study population and sample size.

There was no difference in weight loss between study centres. However there was a greater proportion of male participants from the German (15%) and Australian study centres (16%), compared to UK centres (8%), and a lower baseline BMI on average (− 1.1 kg/m^2^, 95% CI: − 1.5, − 0.6) in German centres, than those from Australian centres (but not UK) [10].

### Procedures

Participants randomised to the CP received free weekly access to community-based, open-group Weight Watchers® meetings for 12 months. This CP promoted a low energy (500 kcal deficit per day) diet based on healthy-eating principles, increased physical activity, self-selected goal setting, weekly weigh-ins, and group behavioural and motivational counselling. Access to a range of internet-based resources for self-monitoring, online forums and information was also provided. The SC group received free weight loss advice based on changes in diet and increases in physical activity from a primary care professional, usually a practice nurse, at their local general practitioner office for 12 months; primary care providers were encouraged to use local and national clinical guidelines for weight management. In all countries, SC participants attended an average of 1 session (GP or nurse consult) per month. In Australia and the UK, CP participants attended 3 CP meetings on average per month; CP participants in Germany attended an average of 2 CP meetings per month [10].

In both groups, body weight (light clothes, no shoes), height, fat mass, waist circumference and blood pressure were measured at baseline, 2, 4, 6, 9 and 12 months using standardised procedures [10]. Physical activity was estimated using pedometers (Weight Watchers International Inc., New York) worn for 7 consecutive days just prior to the baseline visit and at 6 and 12 months after baseline [21].

### Dietary data

All study participants were asked to complete a 4-day food diary, recording all food and beverage consumption in household units, at baseline, 6 and 12 months. Food diaries collected from UK study centres were coded and linked to British food composition tables using the DINO (Diet In, Nutrients Out) in-house programme at MRC Human Nutrition Research [22]. Food diaries collected from Australian participants were coded and linked to Australian food composition tables using Foodworks Professional 2007 (Xyris Software, Brisbane, Australia). Average daily intakes of dietary fibre estimated using the Association of Official Analytical Chemists (AOAC) method in Australia were converted to a non-starch polysaccharide (NSP) equivalent using a conversion factor of 0.75 [23] to enable comparison with UK fibre estimates. Dietary energy-density was estimated as total food energy (excluding beverages) per gram of food consumed (MJ/g per day) [24]. Nutrient densities (protein, total fat, saturated fat, carbohydrate and total sugars) were estimated as g per MJ total energy intake (g/MJ), to enable comparisons of energy-adjusted nutrient intakes, which takes into account changes in energy intake during the intervention. Fibre-density was calculated as g of non-starch polysaccharide fibre (NSP) or equivalent per MJ total energy (g/MJ per day). As is recommended by others, we did not investigate total energy intake as a predictor of weight change, as self-reported energy intake is not a reliable measure when assessing diet and health outcomes [25].

### Statistical analyses

Summary statistics (mean ± SD and *N* (%)) on baseline characteristics were calculated for the CP and SC groups. Distributions of dietary intake over time and by group were described using box plots depicting the median, interquartile ranges, and outliers at baseline, 6 and 12 months; these were also summarised in a supplementary table (Additional file 1: Table S1).

Two sets of analyses were fitted based on linear mixed effects models with a random intercept [26]. The random intercept accounted for the correlation between multiple observations over time taken from each individual.

Firstly, a linear mixed effect model was fitted to the measures of dietary intake (absolute nutrient intakes as g/d and nutrient densities as g/MJ), collected at baseline, 6 and 12 months from each participant. The explanatory variables in the model included intervention group, age at baseline, gender, country, period of observation and an interaction term for period of observation and intervention group. The interaction term allowed us to investigate whether mean changes in dietary intakes between baseline, 6 and 12 months differed by intervention group.

Secondly, a linear mixed effects model was fitted to the measures of body weight collected at baseline, 6 and 12 months for each participant. This model included age, gender, country, physical activity (pedometer steps), intervention group, period of observation, and an interaction term for period of observation and intervention group as explanatory variables. In addition, as the longitudinal effects of changes in dietary intake over time on changes in weight were of most interest, the longitudinal and cross-sectional coefficients for nutrient densities were decomposed [27] and only the longitudinal effect is reported. This measures the impact of a unit change in nutrient intake (or physical activity) on the individual’s weight, and was modelled by including the difference of the explanatory variable into the linear predictor, in addition to the baseline value of the variable. In this model, period of observation, its interaction with intervention group, and the longitudinal effects of the nutrients intakes were time-varying explanatory variables.

The models that best summarise the data were built by comparing hierarchical regression models with different combinations of explanatory variables, after checking for non-linear effects including interactions and quadratic terms. We note that the combination of several correlated measures of nutrient intake and measurement error in a multivariate model may lead to distortion of the underlying associations. However, this problem was largely overcome by the use of nutrient densities (g/MJ) rather than absolute nutrient intakes (g/d) [28]. Furthermore, the separation of longitudinal and cross-sectional effects reduced the correlation among the dietary variables, hence the analyses undertaken yielded highly stable parameter estimates. The models were fitted to the data using maximum likelihood estimation and the significance of individual terms in the model was assessed using Wald tests, plots of residuals were used to check the goodness of fit of the selected models. Finally, to assess patterns of dropout, logistic regression models were used to identify the characteristics of participants who did not provide food diaries after baseline. The statistical analyses were undertaken using R [29] and the package lme4 [30].

## Results

### Baseline characteristics

Baseline anthropometric data were available for all 504 participants and of these, 467 participants completed a food diary (Table 1). Average BMI was 31.6 kg/m^2^ and 88% of participants were female (Table 1). In the CP and SC groups, median proportions of energy from saturated fat (12.7 and 12.5%, respectively) were higher than WHO recommendations at baseline [15, 16] (Additional file 1: Table S1). Average fibre intake was 13.2 g/d (CP) and 13.5 g/d (SC); at least one-quarter below that recommended in the UK (18 g NSP fibre/d) and Australia (18.8–22.5 g NSP fibre/d) at the time of data collection (Additional file 1: Table S1) [31, 32].

**Table 1:** Baseline characteristics by intervention group

	CP			SC		
	*N*	% or mean	(SD)	*N*	% or mean	(SD)
	255			249		
Female	229	90%		213	86%	
Male	26	10%		36	14%	
Age, years	255	47.0	(13.2)	249	47.9	(11.81)
Weight, kg	255	86.2	(11.5)	249	85.9	(11.6)
BMI (kg/m^2^)	255	31.8	(2.6)	249	31.5	(2.4)
Country						
UK	120	47%		116	47%	
Australia	135	53%		133	53%	
Pedometer steps	255	7821	(3546)	249	8199	(3298)
Total energy, kJ/d	239	7573	(2138)	228	7417	(1950)
Total fat, g/d	239	69.1	(24.5)	228	67.1	(22.8)
Saturated fat, g/d	239	26.8	(10.7)	228	25.3	(9.8)
Carbohydrates, g/d	239	206.9	(64.1)	228	200.4	(61.2)
Sugar, g/d	239	88.3	(38.0)	228	88.2	(37.5)
Protein, g/d	239	76	(21.2)	228	75.7	(19.6)
Energy density, MJ/g	239	7.39	(1.50)	228	7.11	(1.58)
Fibre density, g/MJ	239	1.86	(0.55)	228	1.98	(0.69)
Fat density, g/MJ	239	9.0	(1.4)	228	9.0	(1.5)
Saturated fat density, g/MJ	239	3.5	(0.79)	228	3.4	(0.79)
Protein density, g/MJ	239	10.2	(2.1)	228	10.4	(2.1)
Carbohydrate density, g/MJ	239	27.5	(4.0)	228	27.1	(4.4)
Sugar density, g/MJ	239	11.6	(3.8)	228	11.9	(3.9)

Of the 467 participants who completed a baseline food diary, 281 provided a food diary at 6 months (60%) and 209 at 12 months (45%). Those who did not provide a food diary at 12 months tended to be younger (OR = 0.95, 95% CI: 0.94, 0.97), more likely to be from a UK study centre (OR = 3.7, 95% CI: 2.4, 5.7), and more likely to be in the SC group (OR = 2.1, 95% CI: 1.4, 3.2). Characteristics were similar for those participants who did not complete a food diary 6 months after baseline. Gender and BMI were not predictors of food diary completion at 6 months or 12 months.

### Dietary changes at 6 and 12 months after baseline

**Fig. 1:**
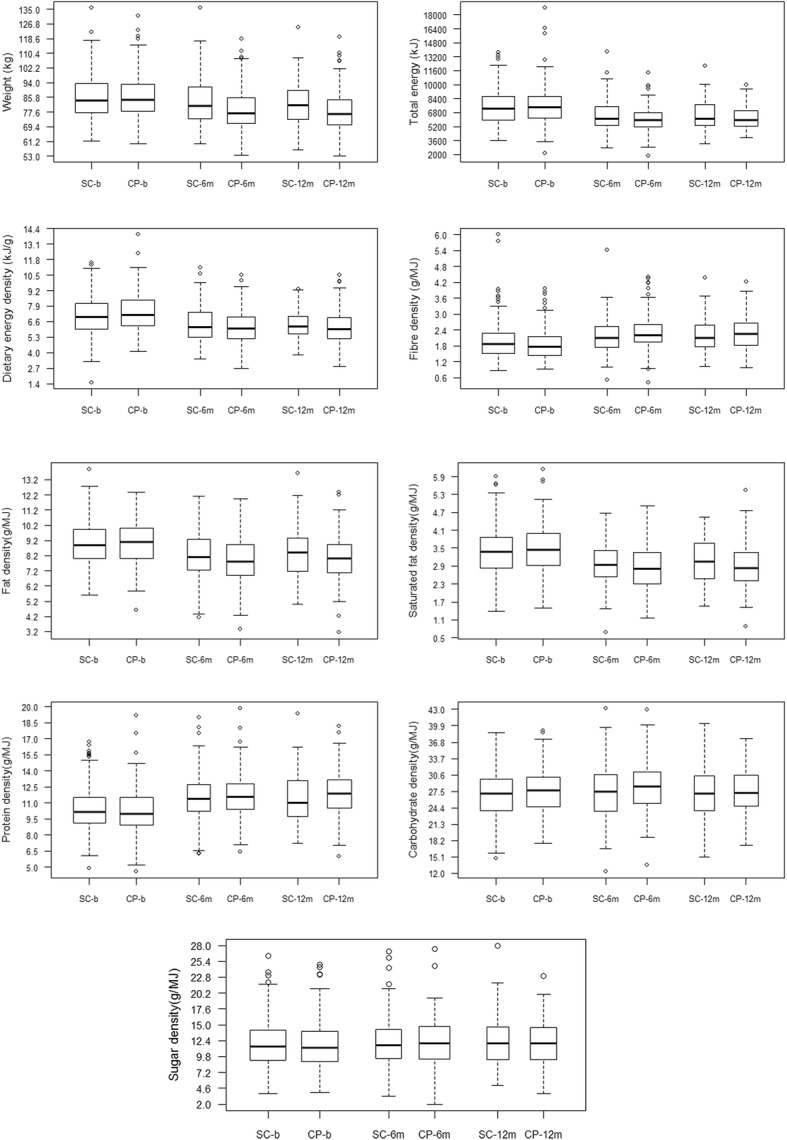
Boxplots of body weight and dietary intakes by group at baseline, 6 and 12 months. Solid line is median, boxes represent quartile (Q) 2 and Q3; dashed bars (whiskers) represent the most extreme upper (Q3 + 1.5(Q3-Q1)) and lower (Q1–1.5*(Q3-Q1)) values; circles show outliers; SC, group assigned to standard care in primary care; CP, group assigned to commercial weight loss programme; b, baseline; 6 m, 6 months; 12 m, 12 months

Figure 1 illustrates median weight and dietary intake for SC and CP groups at baseline, 6 and 12 months. Decreases in median weight, energy intake, dietary energy density, fat density, saturated fat density and increases in median fibre density and protein density were evident over time in both groups.

The overall dietary changes for all participants showed significant reductions in mean energy intake (− 952 kJ/d; 95% CI: − 1263, − 641), total fat (− 12.9 g/d; 95% CI: − 16.7, − 9.1), saturated fat (− 5.7 g/d; 95% CI: − 7.4, − 4.1), total carbohydrate (− 22.2 g/d; 95% CI: − 31.5, − 12.9) and sugars (− 9.7 g/d; 95% CI: − 15.6, − 3.9), as well as dietary energy-density (− 0.64 MJ/g; 95% CI: − 0.89, − 0.39), fat density (− 0.72 g/MJ; 95% CI: − 1.0, − 0.42) and saturated fat density (− 0.40 g/MJ; 95% CI: − 0.54, − 0.25) 6 months after baseline (Table 2). In addition, there were significant increases in mean dietary fibre density (0.14 g/MJ; 95% CI: 0.03, 0.25) and protein density (0.93 g/MJ; 95% CI: 0.53, 1.32) at 6 months (Table 2). Similar changes were observed at 12 months (Table 2). Mean changes in dietary intakes between baseline, 6 and 12 months did not differ significantly by country.

**Table 2:** Adjusted mean changes in dietary intakes over time and between intervention groups

	Energy (kJ/d)	Fat (g/d)	Saturated fat (g/d)	Protein (g/d)	Carbohydrate (g/d)	Sugars (g/d)	
Overall^a^							
6 months v baseline							
*Mean change*	−952	−12.9	−5.7	−3.4	−22.2	− 9.7	
*95% CI*	(− 1263, 641)	(− 16.7, −9.1)	(−7.4, − 4.1)	(−6.7, 0.06)	(− 31.5, − 12.9)	(− 15.6, − 3.9)	
*p-value*^b^	*p* < 0.001	*p* < 0.001	*p* < 0.001	*p* = 0.055	*p* < 0.001	*p* = 0.002	
12 months v baseline							
*Mean change*	−822	−10.5	− 4.7	−3.0	−20.3	− 9.9	
95% CI	(− 1174, − 472)	(− 14.7, − 6.2)	(− 6.6, − 2.9)	(− 6.8, 0.9)	(− 30.8, − 9.8)	(− 16.5, − 3.2)	
*p-value*^b^	*p* < 0.001	*p* < 0.001	*p* < 0.001	*p* = 0.131	*p* < 0.001	*p* = 0.004	
CP versus SC							
6 months v baseline							
*Difference in mean change*	−503	−6.9	− 3.3	−3.1	−11.9	−6.1	
*95% CI*	(− 913, − 93)	(− 11.9, − 1.8)	(− 5.4, − 1.1)	(− 7.6, 1.4)	(− 24.1, 0.3)	(− 13.9, 1.6)	
*p-value*^c^	*p* = 0.017	*p* = 0.008	*p* = 0.003	*p* = 0.173	*p* = 0.057	*p* = 0.120	
12 months v baseline							
*Difference in mean change*	− 465	−7.1	− 3.4	− 0.9	−11.3	−4.4	
*95% CI*	(− 924, − 5)	(− 12.7, − 1.5)	(− 5.8, − 1.1)	(− 6.0, 4.1)	(− 24.9, 2.4)	(− 13.1, 4.3)	
*p-value*^c^	*p* = 0.048	*p* = 0.014	*p* = 0.005	*p* = 0.720	*p* = 0.107	*p* = 0.324	
	Dietary energy density (MJ/g)	Fat density (g/MJ)	Saturated fat density (g/MJ)	Protein density (g/MJ)	Carbohydrate density (g/MJ)	Sugar density (g/MJ)	Fibre density (g/MJ)
Overall^a^							
6 months v baseline							
*Mean change*	−0.64	−0.72	−0.40	0.93	0.76	0.22	0.14
*95% CI*	(− 0.89, − 0.39)	(− 1.0, − 0.42)	(− 0.54, − 0.25)	(0.53, 1.32)	(− 0.001, 1.53)	(− 0.45, 0.88)	(0.03, 0.25)
*p-value*^b^	*p* < 0.001	*p* < 0.001	*p* < 0.001	*p* < 0.001	*p* = 0.051	*p* = 0.525	*p* = 0.015
12 months v baseline							
*Mean change*	− 0.61	− 0.47	−0.31	0.84	0.44	−0.01	0.15
*95% CI*	(− 0.89, − 0.34)	(− 0.80, − 0.13)	(− 0.47, − 0.15)	(0.39, 1.28)	(− 0.42, 1.304)	(− 0.77, 0.74)	(0.02, 0.27)
*p-value*^b^	*p* < 0.001	*p* = 0.006	*p* < 0.001	*p* < 0.001	*p* = 0.318	*p* = 0.975	*p* = 0.021
CP versus SC							
6 months v baseline							
*Difference in mean change*	− 0.48	−0.32	−0.22	0.38	0.18	0.05	0.30
*95% CI*	(− 0.81, − 0.16)	(− 0.71, 0.07)	(− 0.40, − 0.03)	(− 0.14, 0.91)	(− 0.83, 1.19)	(− 0.83, 0.93)	(0.15, 0.44)
*p-value*^c^	*p* = 0.004	*p* = 0.105	*p* = 0.023	*p* = 0.151	*p* = 0.722	*p* = 0.914	*p* < 0.001
12 months v baseline							
*Difference in mean change*	−0.48	−0.47	−0.26	0.65	0.17	0.25	0.26
*95% CI*	(− 0.85, − 0.12)	(− 0.91, − 0.03)	(− 0.47, − 0.05)	(0.07, 1.24)	(− 0.96, 1.30)	(− 0.73, 1.24)	(0.09, 0.42)
*p-value*^c^	*p* = 0.009	*p* = 0.035	p = 0.014	*p* = 0.029	*p* = 0.773	*p* = 0.616	*p* = 0.002

^a^Linear mixed effects regression models for dietary intake collected at baseline, 6 and 12 months, with explanatory variables age, gender, country (all participants from UK and Australian study centres); time, intervention and intervention by time interaction; the difference in mean change is the estimated difference in mean change during the specified follow up period between intervention groups

^b^Probability of rejecting the null hypothesis (mean change = 0) when it is true, based on a Wald test

^c^Probability of rejecting the null hypothesis (difference in mean change = 0) when it is true, based on a Wald test

At 6 months, the CP group showed significantly greater reductions in mean energy intake (− 503 kJ/d; 95% CI: − 913, − 93), total fat (− 6.9 g/d; 95% CI: − 11.9, − 1.8), saturated fat (− 3.3 g/d; 95% CI: − 5.4, − 1.1), saturated fat density (− 0.22 g/MJ; 95% CI: − 0.40, − 0.03), dietary energy density (− 0.48 MJ/g; 95% CI: − 0.81, − 0.16), and greater increases in mean fibre density (0.30 g/MJ; 95% CI: 0.15, 0.44) compared to SC; but no significant differences in change in carbohydrate, sugars or protein (g/d or g/MJ) (Table 2). At 12 months, similar differences were observed between SC and CP, and the CP group additionally showed significantly greater increases in mean protein density (0.65 g/MJ; 95% CI: 0.07, 1.24) than SC (Table 2).

Few dietary differences were observed between study centres, apart from slightly higher mean protein intake (5.4 g/d, 95% CI: 2.3, 8.5) and lower mean sugar intake (− 9.4 g/d, 95% CI: − 15.1, − 3.8) in the Australian centre during the course of the study.

### Dietary changes associated with weight loss

Data on dietary intake and weight change were available for 280 participants at 6 months and 209 at 12 months (including participants Australian and UK centres only). The CP group had greater mean weight loss than the SC group between baseline and 6 months (3.3 kg, 95% CI: 2.2, 4.4) and between baseline and 12 months (3.3 kg, 95% CI: 2.1, 4.5) after adjustment for age, gender, country, and physical activity.

Table 3 shows the estimated regression coefficients from the model of best fit for change in nutrient densities (g/MJ) and change in weight (kg) over the 6 and 12 month follow up periods (regression coefficients are the within-person longitudinal associations between change in dietary intake and weight change). Changes in protein density and fibre density were associated with small but statistically significant weight loss in both intervention groups. A 1 g/MJ increase in protein density over the follow up period was associated with 0.25 kg (95% CI: 0.09, 0.41) greater weight loss (Table 3, model 1). A 1 g/MJ increase in fibre density over the follow up period was associated with a 0.61 kg (95% CI: 0.03, 1.2) greater weight loss. These associations remained after adjustment for physical activity, which was also associated with greater weight loss [21] (Table 3). No other nutrient densities were associated with weight loss. No significant interactions were observed between change in nutrient densities and intervention on weight loss, indicating that the effects of change in protein density and fibre density on weight loss were comparable for CP and SC groups over time.

**Table 3:** Within-person longitudinal associations between change in nutrient density and change in weight between baseline, 6 and 12 months

	*Model 1* ^a^	*Model 2* ^b^
*Explanatory variables*	beta^c^ 95% CI *p*-value^d^	beta^c^ 95% CI *p*-value^d^
Protein density, g/MJ	− 0.254	− 0.256
	− 0.414, − 0.094	−0.415, − 0.097
	*p* = 0.002	*p* = 0.002
Fibre density, g/MJ	− 0.614	−0.676
	−1.202, −0.025	− 1.269, − 0.083
	*p* = 0.041	*p* = 0.026
Physical activity (per 10,000 pedometer steps)		− 1.675
		−2.676, −0.673
		*p* = 0.002

No associations were observed between change in energy density, total fat density, saturated fat density, carbohydrate density or sugar density and change in weight between baseline, 6 and 12 months

^a^Model 1: linear mixed effects regression model of weight collected at baseline, 6 and 12 months, with within-person change in nutrient density between baseline and 6 and 12 month follow up as explanatory variables, and adjusting for age, gender, country, intervention group (SC or CP), time period and intervention group by time interaction

^b^Model 2: as model 1, additionally adjusted for physical activity

^c^Beta coefficient = estimated change in weight (kg) per 1 g/MJ increase in nutrient density or per 10,000 pedometer steps between baseline and end of follow up

^d^Probability of rejecting the null hypothesis (beta = 0) when it is true, based on a Wald test

## Discussion

This longitudinal analysis of a behavioural weight loss intervention has revealed that participants in both intervention groups achieved significant improvements in diet quality. These improvements persisted to 12 months from baseline and included reductions in total energy intake, dietary energy density, total fat (g/d and g/MJ), saturated fat (g/d and g/MJ), carbohydrates (g/d) and sugars (g/d), along with increases in dietary fibre density and protein density (g/MJ). Participants who followed a group-based commercial programme achieved greater improvements in dietary fibre density and protein density (in addition to greater reductions in total energy intake, total fat, and saturated fat) than those in standard care. Regardless of intervention group, only increases in dietary fibre density and protein density were associated with weight loss.

An intention to treat analysis of data from all study centres in this RCT previously showed that those randomised to the CP lost significantly more weight (adjusted difference: 2.77 kg, 95% CI: 2.03, 3.50 kg; based on last observation carried forward) at 12 months after baseline [10]. This association was robust when different methods were used to treat missing weight data, as well as completers-only analyses [10]. The present explanatory analysis restricted to participants from the Australian and UK centres, showed similar differences in weight loss compared to the full trial. Taken together, this suggests that greater increases in dietary fibre density and protein density among participants in the CP group may have contributed to their greater weight loss, compared to SC.

Evidence from observational and experimental studies suggest that increased fibre intake may prevent weight gain, through reductions in appetite and energy intake [19]. A high fibre intake is associated with diets of lower energy density and total energy intake, as fibre increases the weight of food consumed (leading to greater satiety) without providing additional energy [33]. Mechanisms linking dietary fibre with enhanced appetite control include increased food chewing and gut transit times, enhanced gut hormone secretion and fibre fermentation in the colon (leading to greater short chain fatty acids), which may enhance satiety [19]. However, the Scientific Advisory Committee on Nutrition review of Carbohydrates and Health failed to find a consistent association between dietary fibre and body weight in RCTs or cohort studies [20]. The effect on body weight may depend on fibre type, but no clear dose-response relationships for individual fibre types have been identified [19].

Meta-analyses of short and long term (≥12 months) trials indicate that higher protein diets can lead to greater weight loss over the short term and better weight loss maintenance [18]. Dietary protein is proposed to influence key metabolic targets that may enhance weight loss, including sustaining satiety during negative energy balance, maintaining basal energy expenditure despite weight loss, and the sparing of fat free mass [34]. It has been suggested that optimum protein intake for weight loss can be achieved by maintaining absolute protein intake while reducing carbohydrate and fat intake in an energy restricted diet [34]. Consistent with this, we observed no significant changes in absolute protein intake (g/d) and accordingly, reductions in absolute total fat and carbohydrate intakes are likely to have led to the observed increases in dietary protein density (g/MJ), which were linked to weight loss.

Not all of the dietary changes investigated in this study were associated with weight loss, but the observed improvements in diet quality may confer other health benefits. Replacing saturated fats with polyunsaturated fats reduces the risk of coronary heart disease [15, 35, 36] and may help in the prevention and management of Type 2 diabetes [15, 37]. Evidence from RCTs suggests that a reduction in sugar intake may lead to improvements in blood pressure and blood lipids [38], and lower intakes of sugar-sweetened beverages are associated with a decreased risk of Type 2 diabetes [20].

This study adds to the small body of evidence of improvements in energy, macronutrient, and fibre intakes reported by other comparable behavioural weight loss interventions. A 12-week commercial behavioural weight loss intervention for men (the “SHED IT” trial) [4] reported that after 3 and 6 months, men who received self-help resources plus online support for weight loss significantly reduced their intakes of total fat, saturated fat, carbohydrate and sugars, and increased their energy from protein, but made no significant changes in fibre intake [4]. In another Australian web-based commercial weight loss intervention, no significant changes in macronutrient or fibre intake were observed after 12 weeks [5]. The precision and statistical power of these studies may have been reduced however, by using a FFQ to detect dietary changes over a short time period, and by focussing on a relatively small proportion of people who lost ≥5% of their body weight.

The PREMIER trial (*n* = 745) in the US tested two 18-month behavioural lifestyle interventions: one including established recommendations (EST); one including EST plus support to adopt the Dietary Approaches to Stop Hypertension (DASH) diet (EST + DASH); or an advice only group [8]. All groups significantly decreased their mean total energy intake at 6 and 18 months [6]. In addition, both intervention groups significantly decreased mean proportions of energy from total fat, saturated fat, other fats, and carbohydrate, with the greatest changes in the EST + DASH group. The EST + DASH group also significantly increased mean fibre intake and energy from protein [6].

Following on from the PREMIER trial, the US Weight Loss Maintenance Study included an initial 6 month intensive behavioural weight loss intervention (Phase I) in which all participants (*n* = 1685) were encouraged to follow the DASH diet [7]. Dietary changes were reported for 828 participants who had lost at least 4 kg body weight at the end of Phase 1. These changes included significant reductions in total energy intake and energy from fat, and significant increases in fibre intake and energy from carbohydrate and protein [7].

Not all studies report the relationship between changes in dietary intake and weight loss. However in the SHED IT trial, those who lost at least 5% of their baseline weight (*n* = 49, 18%) reported significantly greater reductions in total energy, energy from sugars, and greater increases in energy from protein (but not fat, carbohydrate, or fibre) compared with unsuccessful completers. In the PREMIER trial, a secondary analysis including 501 participants who were overweight or obese at baseline (68% obese) found that a 1% decrease in energy from total fat between baseline and 6 months was associated with a 0.06% decrease in weight (*p* < 0.05) [8]. Unlike the present study, no associations were observed between weight change and energy from protein, and dietary fibre was not examined (no associations were observed between weight loss and total energy or total carbohydrate intake) [8]. In the initial 6 month intensive behavioural weight loss intervention (Phase 1) of the Weight Loss Maintenance study, it was found that substituting dietary fat with protein or carbohydrate, or substituting carbohydrate with protein, was associated with greater weight loss at 6 months. Unlike our present study, fibre intake was not associated with greater weight loss [7]. This study benefited from a large sample size, however, by restricting the analysis to only those participants who lost at least 4 kg weight and who completed a FFQ at four time points, the likelihood of selection bias was increased.

Collectively, these studies indicate that structured dietary programmes can achieve specific improvements in dietary intake which may enhance weight loss. An increase in energy from protein was frequently linked with weight loss but the effects of total fat, carbohydrates and fibre are less clear.

Unlike earlier comparable studies that used an FFQ to assess dietary changes [4, 5, 7] the present study included 4-day food diaries that were linked to country-specific food composition data to provide detailed information on food intake at three time points over 12 months. The food diary or record is an ideal dietary assessment as it collects rich data on food choice, portion size and cooking methods at the meal-level, which are highly relevant to understanding eating behaviours conducive to weight loss. Furthermore, food diaries can capture dietary intake over a short time period and are well suited as repeated dietary assessments over the duration of a trial. Whereas, a FFQ does not collect detailed data at the meal level and by design, is better suited for summarising usual dietary intake over an extended time period, typically 1 year. However, it is recognised that dietary under-reporting is an issue with all dietary assessments that are based on self-report [25]. We did not attempt to adjust for dietary under-reporting because all respondents were expected to be purposefully reducing their energy intake in this weight loss intervention, and current methods to identify dietary under-reporting assume energy balance. We did not use a baseline indicator of under-reporting and assume this remained constant over the course of the trial, as this would be likely to incorporate additional misclassification error. However, the use of nutrient densities in this analysis would have mitigated the impact of dietary under-reporting to some degree [25]. Furthermore, our analysis examined individual-level changes in dietary intake over time (using longitudinal models), rather than comparing group-level mean intakes, as is commonly reported. Including data on dietary intake and weight up until 12 months after baseline is another advantage, as many published studies of this nature are brief interventions averaging 12 weeks in duration or report associations up to 6 months [5, 7, 8].

High participant dropout rates are very common in weight loss trials [39] and a high dropout rate (non-completion of dietary assessments) was evident in this secondary analysis. As changes in weight and/or dietary intake may have differed in completers and non-completers, a biased study population may have resulted from participant dropout, reducing the generalisability of our findings. Gender bias is also not uncommon in weight loss interventions. Our study population was predominantly female (88%) and therefore, the results are less generalisable to men. However, the use of multivariate, longitudinal models to examine dietary changes and their relationships with weight loss at three time points is a major study strength. Mixed effects models utilised all available follow up data rather than limiting the analyses to those who completed all follow ups only. The multivariate models analysed changes in dietary intake taking account of baseline values and individual level changes while adjusting for relevant confounders. Furthermore, in our analysis we separated the cross-sectional from the longitudinal associations between changes in dietary intake and weight loss.

## Conclusions

Participation in a behavioural weight loss intervention leads to improvements in diet quality, and these were significantly greater among those attending a group-based commercial programme than those receiving support provided in primary care. Weight loss in both groups was associated with increased dietary fibre density and protein density, which occurred concurrently with reductions in absolute intakes of fat, carbohydrate and total energy, suggesting these are important dietary targets for weight loss.

## Additional file

Additional file 1: **Table S1**. Characteristics by intervention group (SC, standard care; CP, commercial programme) at baseline, 6 and 12 months. (DOCX 26 kb)

## Abbreviations

AOAC: Association of Official Analytical Chemists
BMI: Body mass index
CP: Commercial programme (intervention group)
FFQ: Food frequency questionnaire
RCT: Randomised controlled trial
SC: Standard care (intervention group)
