# Supplementary material for: Greater improvements in diet quality among overweight participants following a group-based commercial weight loss programme than those receiving support to lose weight in primary care
Source: Nutr J. 2018 Jul 4;17:64. doi: 10.1186/s12937-018-0370-x (PMC6032789; doi:10.1186/s12937-018-0370-x)
Supplement: Supplementary file 1 — Table S1. Characteristics by intervention group (SC, standard care; CP, commercial programme) at baseline, 6 and 12 months. (DOCX 26 kb) [file 12937_2018_370_MOESM1_ESM.docx]

**Additional file 1: Table S1. Characteristics by intervention group (SC, standard care; CP, commercial programme) at baseline, 6 months and 12 months**

|  |  | Baseline |  |  |  | 6 months |  |  |  | 12 months |  |  |
| --- | --- | --- | --- | --- | --- | --- | --- | --- | --- | --- | --- | --- |
|  | SC |  | CP |  | SC |  | CP |  | SC |  | CP |  |
|  | n | % or median (IQR) | n | % or median (IQR) | n | % or median (IQR) | n | % or median (IQR) | n | % or median (IQR) | n | % or median (IQR) |
| Male | 31 | 13.6 | 24 | 10.0 | 19 | 16.2 | 15 | 9.1 | 14 | 16.3 | 13 | 10.6 |
| Female | 197 | 86.4 | 215 | 90.0 | 98 | 83.8 | 149 | 90.9 | 72 | 83.7 | 110 | 89.4 |
| UK centre | 102 | 44.7 | 109 | 45.6 | 37 | 31.6 | 68 | 41.5 | 24 | 27.9 | 38 | 30.9 |
| Australian centre | 126 | 55.3 | 130 | 54.4 | 80 | 68.4 | 96 | 58.5 | 62 | 72.1 | 85 | 69.1 |
| Age, y | 228 | 49.1 (17.1) | 239 | 46.2 (20.4) | 117 | 53.8 (14.4) | 164 | 49.8 (18.3) | 86 | 54.9 (12.4) | 123 | 51.5 (19.5) |
| Weight, kg | 228 | 84.0 (15.7) | 239 | 84.3 (15.3) | 117 | 81.1 (17.7) | 163 | 76.4 (12.2) | 86 | 80.5 (16) | 123 | 77.3 (15.9) |
| BMI (kg/m2) | 228 | 31.4 (3.3) | 239 | 31.7 (3.8) | 117 | 30.4 (3.7) | 163 | 29.1 (3.9) | 86 | 30.5 (3.4) | 123 | 29.3 (4.8) |
| Total energy, kJ | 228 | 7279 (2694) | 239 | 7397 (2564) | 117 | 6065 (2171) | 164 | 5915 (1673) | 86 | 6095 (2442) | 123 | 5978 (1797) |
| Total fat, g/d | 228 | 65.5 (30.8) | 239 | 66 (32.5) | 117 | 48.5 (21.6) | 164 | 45.2 (20.9) | 86 | 52.3 (28.1) | 123 | 50.1 (20.1) |
| Saturated fat, g/d | 228 | 24 (13) | 239 | 25.1 (13.7) | 117 | 18.3 (8.2) | 164 | 16.4 (8.9) | 86 | 18.8 (11.8) | 123 | 18 (9.6) |
| Protein, g/d | 228 | 73.2 (24.3) | 239 | 75.4 (25.3) | 117 | 71.3 (23) | 164 | 68.8 (20.3) | 86 | 72 (25.5) | 123 | 70.7 (24.2) |
| Carbohydrate, g/d | 228 | 194.5 (74.3) | 239 | 200.3 (81.2) | 117 | 165.2 (57.3) | 164 | 168.8 (62.3) | 86 | 162.5 (61.1) | 123 | 169.7 (52.9) |
| Sugars, g/d | 228 | 79.9 (41.8) | 239 | 82.3 (51.2) | 117 | 70.9 (37.6) | 164 | 68.1 (33.1) | 86 | 71.8 (37.3) | 123 | 72.3 (37.1) |
| Fibre*, g/d | 228 | 13.5 (6.1) | 239 | 13.2 (5.8) | 117 | 13.4 (5.2) | 164 | 13.6 (5.5) | 86 | 12.8 (3.9) | 123 | 13.7 (5.9) |
| Energy density, kJ/g | 228 | 7.0 (2.2) | 239 | 7.1 (2.2) | 117 | 6.1 (2.1) | 164 | 6.1 (1.8) | 86 | 6.2 (1.5) | 123 | 6.0 (1.8) |
|  |  | Baseline |  |  |  | 6 months |  |  |  | 12 months |  |  |
|  | SC |  | CP |  | SC |  | CP |  | SC |  | CP |  |
|  | n | % or median (IQR) | n | % or median (IQR) | n | % or median (IQR) | n | % or median (IQR) | n | % or median (IQR) | n | % or median (IQR) |
| Total fat density, g/MJ | 228 | 8.9 (1.9) | 239 | 9.1 (2) | 117 | 8.1 (2) | 164 | 7.8 (2) | 86 | 8.4 (2.2) | 123 | 8.0 (1.9) |
| Saturated fat density, g/MJ | 228 | 3.4 (1.0) | 239 | 3.4 (1.1) | 117 | 2.9 (0.9) | 164 | 2.8 (1) | 86 | 3.0 (1.2) | 123 | 2.8 (1) |
| Protein density, g/MJ | 228 | 10.2 (2.4) | 239 | 9.9 (2.6) | 117 | 11.4 (2.5) | 164 | 11.5 (2.4) | 86 | 11.0 (3.4) | 123 | 11.9 (2.7) |
| Carbohydrate density, g/MJ | 228 | 27.2 (6.0) | 239 | 27.7 (5.6) | 117 | 27.4 (6.9) | 164 | 28.5 (6) | 86 | 27.1 (6.6) | 123 | 27.3 (5.9) |
| Sugar density, g/MJ | 228 | 11.4 (5.0) | 239 | 11.2 (5) | 117 | 11.7 (4.7) | 164 | 12 (5.3) | 86 | 12.0 (5.4) | 123 | 12.0 (5.3) |
| Fibre density, g/MJ | 228 | 1.9 (0.8) | 239 | 1.8 (0.7) | 117 | 2.1 (0.8) | 164 | 2.2 (0.7) | 86 | 2.1 (0.8) | 123 | 2.3 (0.9) |
| Energy from total fat (%) | 228 | 32.8 (6.8) | 239 | 33.0 (7.5) | 117 | 30.0 (8.0) | 164 | 28.8 (6.8) | 86 | 31.4 (8) | 123 | 29.0 (7.0) |
| Energy from saturated fat (%) | 228 | 12.5 (3.8) | 239 | 12.7 (4) | 117 | 10.9 (3.3) | 164 | 10.4 (3.9) | 86 | 11.3 (4.4) | 123 | 10.5 (3.6) |
| Energy from protein (%) | 228 | 17.5 (4.4) | 239 | 17.3 (4.5) | 117 | 19.5 (5.0) | 164 | 19.8 (4.3) | 86 | 18.9 (6) | 123 | 20.3 (4.8) |
| Energy from carbohydrate (%) | 228 | 43.5 (9.4) | 239 | 44.3 (8.3) | 117 | 44 (11.5) | 164 | 45.6 (9.3) | 86 | 43.4 (10.8) | 123 | 44.5 (9.5) |
| Energy from sugars (%) | 228 | 18.5 (8.4) | 239 | 18.0 (8.0) | 117 | 18.8 (7.8) | 164 | 19.6 (8.5) | 86 | 19.3 (8.8) | 123 | 19.8 (8.5) |

*NSP or equivalent converted from AOAC fibre
